# The CAG promoter maintains high‐level transgene expression in HEK293 cells

**DOI:** 10.1002/2211-5463.13029

**Published:** 2020-12-03

**Authors:** Yuanyuan Dou, Yan Lin, Tian‐yun Wang, Xiao‐Yin Wang, Yan‐long Jia, Chun‐peng Zhao

**Affiliations:** ^1^ Department of Biochemistry and Molecular Biology Xinxiang Medical University China; ^2^ Henan Collaborative Innovation Center of Molecular Diagnosis and Laboratory Medicine Xinxiang China

**Keywords:** CAG, expression vector, HEK293F cells, promoter, transgene expression

## Abstract

The vast majority of therapeutic recombinant proteins are produced in mammalian cell lines. However, proteins generated in nonhuman cell lines, such as Chinese hamster ovary (CHO) cells, are decorated with human‐like glycan structures that differ from those of human cells, and these may induce immunogenic responses in human cells. Human embryonic kidney cells (HEK293F) are also extensively used as hosts for the expression of recombinant therapeutic proteins, but their utility is limited by the low expression of transgenes in these cells. Here, we investigated recombinant protein expression from eight frequently used promoters in transfected HEK293F cells. The expression levels and stability of the transgenes were evaluated by flow cytometry and qRT‐PCR. The most efficient expression (in terms of both mRNA and protein yields) was achieved using a cytomegalovirus (CMV) major immediate‐early enhancer combined with the chicken beta‐actin promoter (CAG) promoter, as compared to all other tested promoters under both transient and stable transfection conditions. In addition, application of mild hypothermia (i.e., 33 °C) after transfection improved the positive effect of the CMV enhancer fused to the chicken beta‐actin promoter (CAG promoter) on enhanced green fluorescent protein (eGFP) expression. Although the temperature sensitivity of the CMV promoter is greater than that of CAG promoter, recombinant protein levels were still highest when expression was driven by the CAG promoter. When eGFP was replaced with hepatitis B surface antigen, the CAG promoter still showed the highest transgene expression. In conclusion, our data show that the CAG promoter is a strong promoter for recombinant protein expression in HEK293F cells.

## Graphical abstract

Through flow cytometry and qPCR technology, the promoter vector for high expression of eGFP protein in HEK293F cells was optimized and verified with hepatitis B surface antigen. The CAG promoter vector can not only effectively increase and maintain the expression level of recombinant proteins, but also increase the expression of transgenes under low temperature conditions.

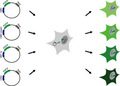

**Abbreviations**
CAG promoter: cytomegalovirus (CMV) enhancer fused to the chicken beta‐actin promoter
CHEF‐1α: Chinese hamster elongation factor‐1α
CHO: Chinese hamster ovary
CMV: cytomegalovirus
eGFP: enhanced green fluorescent protein
HBsAg: hepatitis B surface antigen
HEF‐1α: human elongation factor‐1α
HEK: human embryonic kidney
MAR: matrix attachment region
MFI: median fluorescence intensity
PGK: phosphoglycerate kinase
POI: protein of interest
SV40: Simian virus 40

Approximately 80% of recombinant therapeutic proteins and antibodies are produced in mammalian cells due to the requirement for post‐translational modifications that are similar to those in human cells [1, 2, 3, 4]. Several cell lines are commonly used to produce recombinant therapeutic proteins, including Chinese hamster ovary (CHO) cells, human embryonic kidney (HEK)293 cells, and SP2/0 and NS0 mouse myeloma cells [5]. However, recombinant proteins produced in nonhuman mammalian cells are decorated with two human‐like glycan structures that differ from those of human cells, namely N‐glycolylneuraminic acid and galactose‐alpha‐1,3‐galactose group. In human cells, these proteins can be immunogenic, as antibodies are sometimes produced against these two glycan structures [6, 7]. This immunogenicity can be avoided by producing recombinant proteins in HEK293F cells [8, 9]. In addition, it is easy to rapidly produce recombinant proteins through transient expression using HEK293F cells [10, 11, 12, 13].

Although HEK293 cells have some merits in terms of the production of recombinant therapeutic proteins, low protein yield is an issue that needs to be resolved. One effective strategy to improve transgene expression levels is optimization of the expression vector components [14, 15, 16, 17]. The human elongation factor‐1α (HEF‐1α) [18, 19], mouse phosphoglycerate kinase (PGK), and CMV promoter [20] as well as other natural promoters have been used to drive recombinant protein expression in mammalian cell lines [21]. However, the yield of transgenes driven by the CMV promoter decreased with culture time due to complex transcriptional silencing and DNA methylation issues [22, 23]. In addition to natural promoters, artificial promoters have also been used to promote stable transgene expression. For example, the cytomegalovirus (CMV) enhancer fused to the chicken beta‐actin promoter (CAG) promoter is a robust artificial construct composed of a CMV enhancer combined with the chicken‐actin promoter [24].

Studies have shown that the use of low culture temperatures is an effective strategy for improving the expression of foreign proteins [25]. Reducing the cell culture temperature after transfection can further enhance the yields of recombinant proteins in CHO cells [26, 27, 28]. Although the mechanism underlying this phenomenon is poorly understood, at low temperatures, the S100 calcium‐binding protein A6 promoter showed higher expression levels than the Simian virus 40 (SV40) promoter [29]. In HEK293F cells, lower culture temperatures have a similar effect on transgene expression [30].

Here, we investigated the impact of eight promoters, CAG, HEF‐1α, CMV mutant, Chinese hamster elongation factor‐1α (CHEF‐1α), CAG enhancer, mouse CMV, PGK, and CMV, on transgene expression in HEK293F cells. We further explored the effect of mild low‐temperature culture on the expression of recombinant protein in transfected HEK293 cells.

## Materials and methods

### Vector construction

**Fig. 1:**
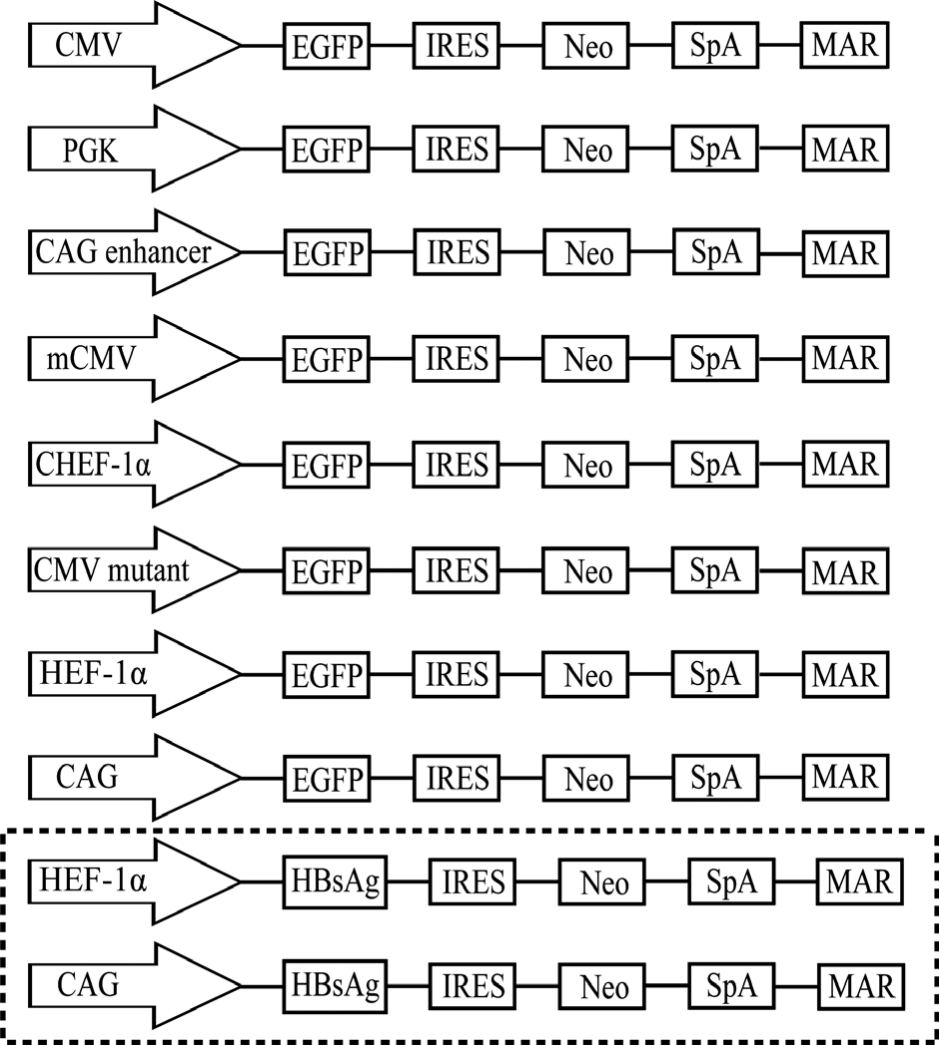
Schematic representation of vectors containing different promoters, CMV mutant, and CAG enhancer element. The constructed vectors containing different elements were used in this study.

The pEGFP‐eGFP‐matrix attachment region (MAR) plasmid, which contains the CMV promoter and MAR element, was previously constructed in our laboratory [31, 32, 33, 34, 35, 36]. The CAG (GenBank accession no: GU299216.1, position 3–1664), HEF‐1α (accession no: AY188393.1, position 10964–12623), CMV mutant, CHEF‐1α (accession no: KY447299.1, position 12–1346), CAG enhancer (accession no: AJ575208.1, position 100–386), mouse CMV (accession no: KT343252.1, position 1103–1625), and PGK (accession no: KJ175229.1, position 641–1195) [37] promoters were synthesized and used to replace the CMV promoter in the pEGFP‐eGFP vector (Fig. 1).

To evaluate the effect of each promoter on the expression of a protein of interest (POI), the enhanced green fluorescent protein (eGFP) gene was replaced with hepatitis B surface antigen (HBsAg) (accession no: ABY65392.1) in vectors containing the CMV enhancer and CHEF1‐α (Fig. 1). A codon‐optimized HBsAg gene was synthesized and then inserted into the pIRES‐neo vector.

### Cell culture and transfection

HEK293F cells were cultured in Dulbecco's Modified Eagle's medium/12 (ProteinEasy, Xinxiang, China) supplemented with 10% FBS (ProteinEasy) and 1% penicillin and streptomycin (ProteinEasy) in a humidified incubator at 37 °C with 5% CO_2_. Cells were seeded at a density of 2 × 10^5^ cells/well into 24‐well plates and incubated overnight. The next day, when the cells reached 80% confluence, they were transfected with the vectors mentioned above with Lipofectamine 2000 Transfection Reagent (Invitrogen, Carlsbad, CA, USA) according to the manufacturer's instructions. Approximately 48 h after transfection, the cells were screened and cultured in medium supplemented with blasticidin (Sangon Biotech, Shanghai, China) for ~ 2 weeks and then collected for analysis.

To further increase the expression of eGFP, cells were moved from 37 to 33 °C after transient transfection. Seven days later, eGFP was detected by flow cytometry to analyze the protein expression yield under mild, low‐temperature conditions. High‐expression vectors containing HBsAg were transfected in the same way as the vectors containing eGFP. At 48 h post‐transfection, the cultures were centrifuged to pellet cells, which were transferred to serum‐free medium (ProteinEasy) in a CO_2_ isothermal shaking incubator. After 5 days, the supernatant was collected by centrifugation, and the following analysis was performed.

### Transfection efficiency and transient expression analysis

Transfection efficiency was assessed by eGFP antibody labeling. At 48 h after transfection, HEK293F cells were resuspended with a prechilled GFP antibody suspension to determine the percentage of cells that were successfully transfected, that is, the transfection efficiency, which was expressed as the ratio of eGFP‐positive cells to the total cells. To accurately analyze transient expression levels, HEK293F cells were collected, and the green fluorescence of the cells was observed at 200× magnification using a 530/15 bandpass filter at an emission wavelength of 530 nm and an excitation length of 480 nm, and the median fluorescence intensity (MFI) of eGFP was evaluated using a Guava EasyCyte 8HT flow cytometer (Millipore Sigma, Darmstadt, Germany). Fluorescence intensity was positively correlated with the promoter's driving effect on the expression of eGFP protein.

### Stable expression analysis

At 48 h post‐transfection, cells were passaged and the transfectants were stabilized by incubation with 20 μg·mL^−1^ blasticidin. After 14 and 30 days of incubation, the fluorescence intensity of the cells was observed under a fluorescence microscope, and eGFP expression was assessed by flow cytometry using a Guava EasyCyte™ 8HT flow cytometer and flow jo software (version 7.6; Tree Star, Ashland, OR, USA).

At 48 h after transfection, when the HEK293F cells transfected with the HBsAg‐containing vectors reached 90% confluence, the cells were harvested with 0.25% trypsin/EDTA and transferred to protein‐free, serum‐free HEK293F suspension medium (ProteinEasy). The appropriate concentration of blasticidin was added, and the cells were placed in a 125 mL Corning shake flask (Sigma # 431255) containing 20 mL of culture medium and incubated for 14 days. A population of stably transfected cells was cultured, and when the cell density reached 8 × 10^6^/mL, the supernatant was collected to measure the expression levels of HBsAg. All experiments were performed in triplicate.

### RNA extraction and quantitative real‐time PCR

Total RNA was extracted from each vector‐transfected cell line and was reverse transcribed using a cDNA Synthesis Kit (ProteinEasy) according to the manufacturer’s instructions. The PCR primers used for GAPDH [38] were as follows: (forward, 5′‐GAGAGACCCTCACTGCTG‐3′ and reverse, 5′‐GATGGTACATGACAAGGTGC‐3′; HBsAg forward, 5′‐TTGGCCAAAATTCG‐CAGTCC‐3′ and reverse, 5′‐TGAGGCATAGCAGCAGGATG‐3′. PCR was carried out according to standard procedures, and qRT‐PCR was performed in a PikoReal™ 2.2 Real‐Time PCR system (Thermo Scientific, Waltham, MA, USA).

### Western blot analysis

When the cell density in the shake flask reached 6 × 10^6^/mL, the supernatant was collected, and HBsAg expression was analyzed by immunoblotting, and a small amount of each sample was saved for ELISA. The supernatant was mixed with 5× SDS sample buffer and boiled for 10 min. Then, a 10 µL aliquot of each sample was separated by SDS/PAGE on a 15% gel and transferred to a nitrocellulose membrane by electroblotting. The membrane was incubated with a 1 : 1000 dilution of anti‐Hepatitis B virus surface antigen antibody (ab9193; Abcam Inc., Cambridge, MA, USA) followed by a 1 : 5000 dilution of a secondary goat anti‐horse antibody conjugated to alkaline phosphatase (Jackson Immuno Research Laboratory, West Grove, PA, USA). Densitometric analysis was performed using imagej v2.1.4.7 software (National Institutes of Health, Bethesda, MD, USA).

### ELISA

The cell supernatant was collected, and HBsAg production was determined by using an ELISA kit according to the manufacturer’s instructions (Meimian Biotechnology, Yancheng, Jiangsu, China). Then, the absorbance values of the treated Microlon ELISA plates were detected with a microplate reader (Synergy HT; BIOTEK, Broadview, IL, USA).

### Statistical analysis

All experimental data were analyzed using spss 18.0 software (SPSS Inc., Chicago, IL, USA). Data are reported as mean ± SD. All experiments were performed three times, and *t*‐tests were used for comparisons. Differences with *P* values < 0.05 were considered statistically significant.

## Results

### Transfection efficiency and transient transgene expression

**Fig. 2:**
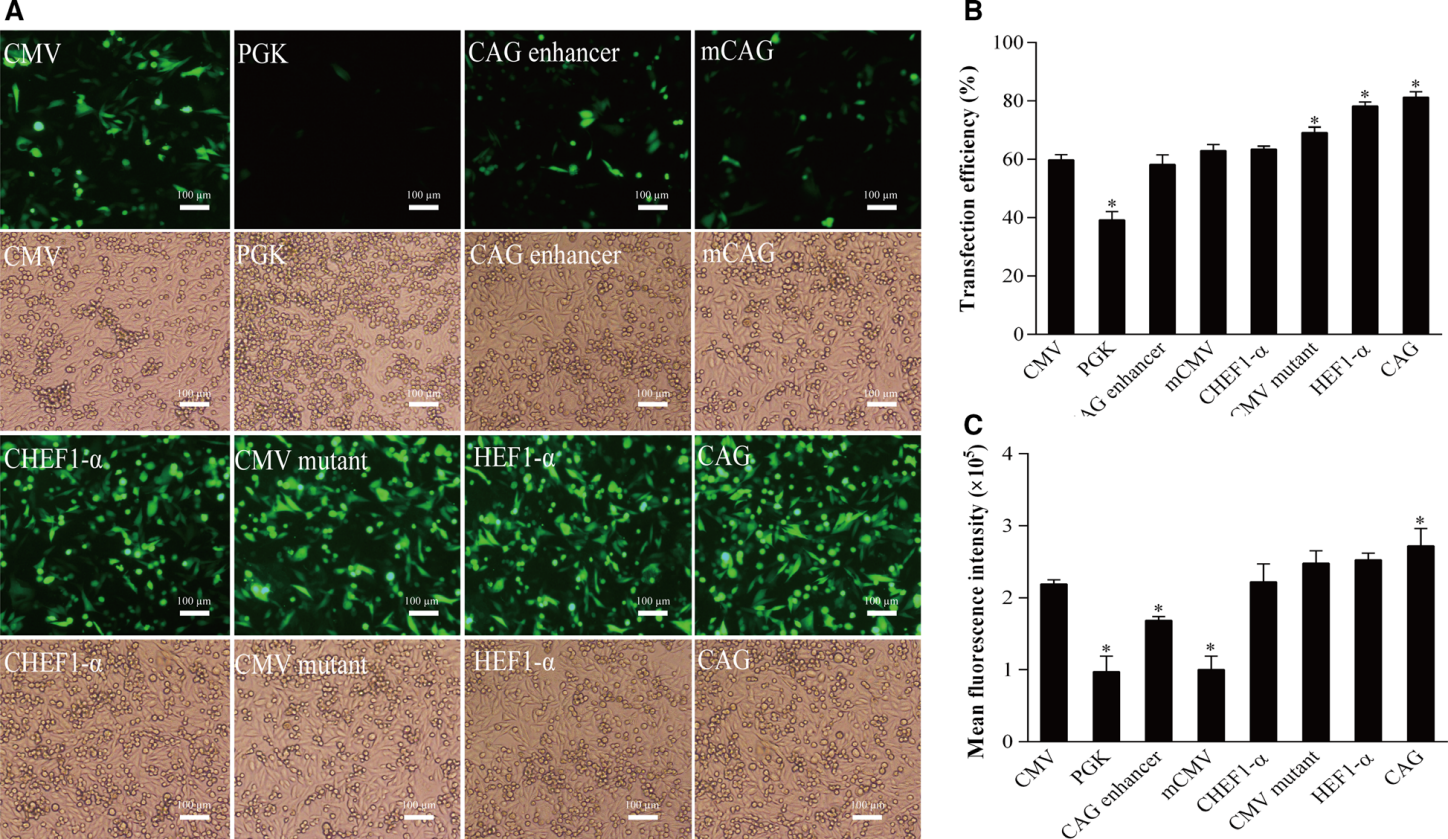
The transient expression of eGFP of the vectors containing different promoter elements in HEK293F cells. (A) The fluorescence spectrum of eGFP was observed after transfection for 48 h under the fluorescence microscope, scale bars: 100 μm. (B) Transfection efficiency. Transfection efficiency was detected using eGFP antibody analysis. (C) Transient eGFP expression was measured by flow cytometry 48 h post‐transfection. The eGFP MFI of transient transfected cells containing different promoters were detected by flow cytometry. All the experiments were repeated three times. SEM is indicated (Student's *t*‐test, *P < 0.05).

The transfection efficiency of the eight promoter constructs in HEK293 cells was evaluated. Vectors containing the CAG promoter had the highest transfection efficiency (81.3%), followed by HEF1‐α (75.1%), CMV mutant (67.7%), CHEF1‐α (63.4%), and CMV (59.1%; Fig. 2B). The lengths of the CAG, HEF1‐α, CMV mutant, CHEF‐1α, CAG enhancer, mouse CMV, PGK, and CMV promoter inserts were 1662, 1659, 589, 1335, 287, 523, 555, and 589 bp, respectively. Given that each vector contains different cis‐acting elements, their lengths are different. Therefore, we evaluated the correlation between vector length and transfection efficiency. The results showed that the transfection efficiency did not vary with the size of the vectors, indicating that the length of the promoter is not the most crucial factor affecting transfection efficiency.

Next, the transient transgene expression levels of eGFP from the eight promoters constructs was evaluated. At 48 h after transfection, the eGFP fluorescence intensity of HEK293F cells transfected with vectors containing eGFP under the control of various promoters was observed under a fluorescence microscope (Fig. 2A). The results indicated that the CAG, HEF‐1α, CHEF‐1α, and CMV mutant promoters enhanced GFP transgene fluorescence intensity compared to that from the CMV promoter. Of the eight tested promoters, the CAG promoter showed the strongest fluorescence. The MFI was detected by flow cytometry, and the results indicated that compared with the CMV promoter, the fluorescence enhancement was highest for the CAG promoter (Fig. 2C), followed by HEF1‐α, CMV mutant, CHEF‐1α, and CAG enhancer. In addition, the expression levels from the mouse CMV and PGK vectors were lower than that from the CMV promoter. The driving effect of the CAG promoter on transgene expression was significantly higher than that of the other seven promoters.

### Recombinant protein expression levels in stably transfected cells

**Fig. 3:**
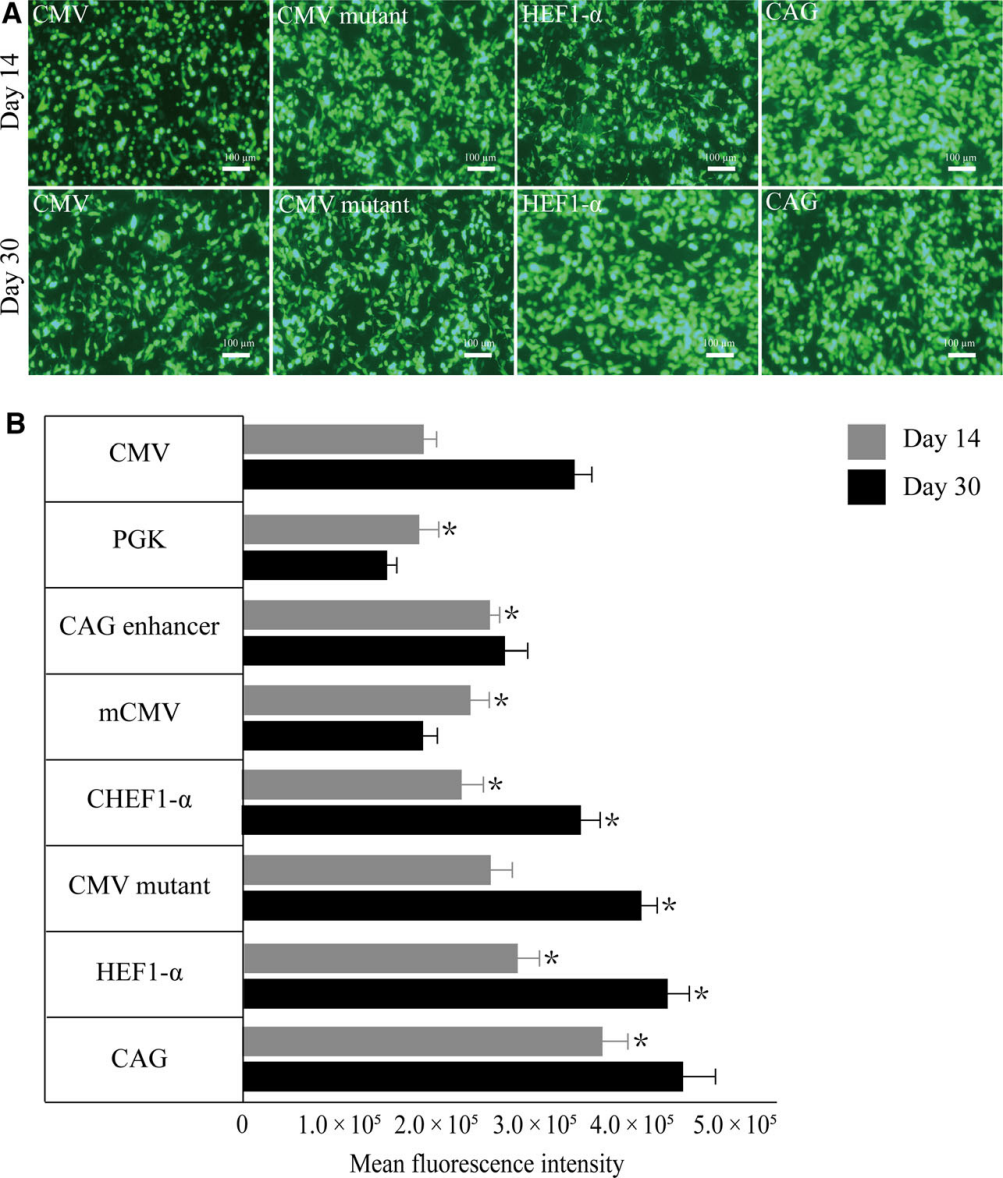
Cells were collected 14 and 30 days after transfection, respectively, and eGFP expression levels were detected by fluorescence microscopy and flow cytometry. (A) The fluorescent expression level was detected at 14 and 30 days after transfected with four high‐expression promoter: CAG, HEF1‐α, CMV mutant, and CMV, scale bars: 100 μm. (B) Flow cytometry analysis of eGFP stable expression at 14 and 30 days after transfection, and the eGFP MFI was normalized to CMV promoter. All the experiments were repeated three times. SEM is indicated (Student’s *t*‐test, *P < 0.05).

We tested different concentrations of blasticidin to select the appropriate concentration for screening stably transfected HEK293F cells and found that 20 μg·mL^−1^ was an effective concentration. After 14 days of blasticidin selection, stably transfected cell lines were obtained. On the 14th and 30th days (Fig. 3A), stable eGFP expression from each promoter vector was detected by fluorescence microscopy and flow cytometry. The results of the fluorescence analysis indicated that CAG and HEF1‐α showed the highest fluorescence after 14 and 30 days of blasticidin selection, followed by CMV mutant and CMV, while fluorescence from CHEF1‐α, CAG enhancer, mouse CMV, and PGK was weaker. The flow cytometry results showed that the CAG, HEF1‐α, CMV mutant, and CHEF1‐α promoters had a strong effect on protein expression, which was superior to that of the CAG enhancer, mouse CMV, PGK, and CMV promoters. Among them, the CAG and HEF1‐α promoters had the highest eGFP levels (Fig. 3B). Although blasticidin selection takes longer, transgene expression levels are higher. However, this was not the case for the mouse CMV and PGK promoters, which may be due to the weak driving effect of the promoter on transgene expression. Selection with blasticidin caused the cells to lose fluorescence, which is not conducive to stable recombinant protein expression.

### eGFP mRNA expression

**Fig. 4:**
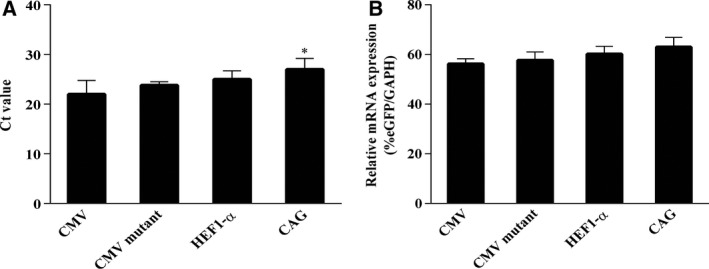
eGFP expression at mRNA level in cells transfected with CAG, HEF‐1α, CMV mutant, and CMV promoters at 14 days after transfection. (A) The mRNA expressions of report gene (eGFP) and internal reference gene (GAPDH) were measured by qRT‐PCR. (B) The mRNA expression levels of cells were calculated using the percentage of eGFP/GAPDH. All the experiments were repeated three times. SEM is indicated (Student’s *t*‐test, *P < 0.05).

The mRNA expression level can reflect the recombinant protein expression level. After transfecting the cells with vectors containing the CAG, HEF‐1α, CMV mutations, and CHEF‐1α promoter constructs, quantitative real‐time PCR was performed. The Ct values were determined (Fig. 4A), and mRNA expression levels were measured. The CAG promoter showed the highest eGFP mRNA expression levels, followed by the HEF‐1α, CMV mutation, and CHEF‐1α promoters (Fig. 4B). The eGFP mRNA expression levels were consistent with the protein expression levels, but the fold increase differed.

### The effects of mild low temperature on protein expression

**Fig. 5:**
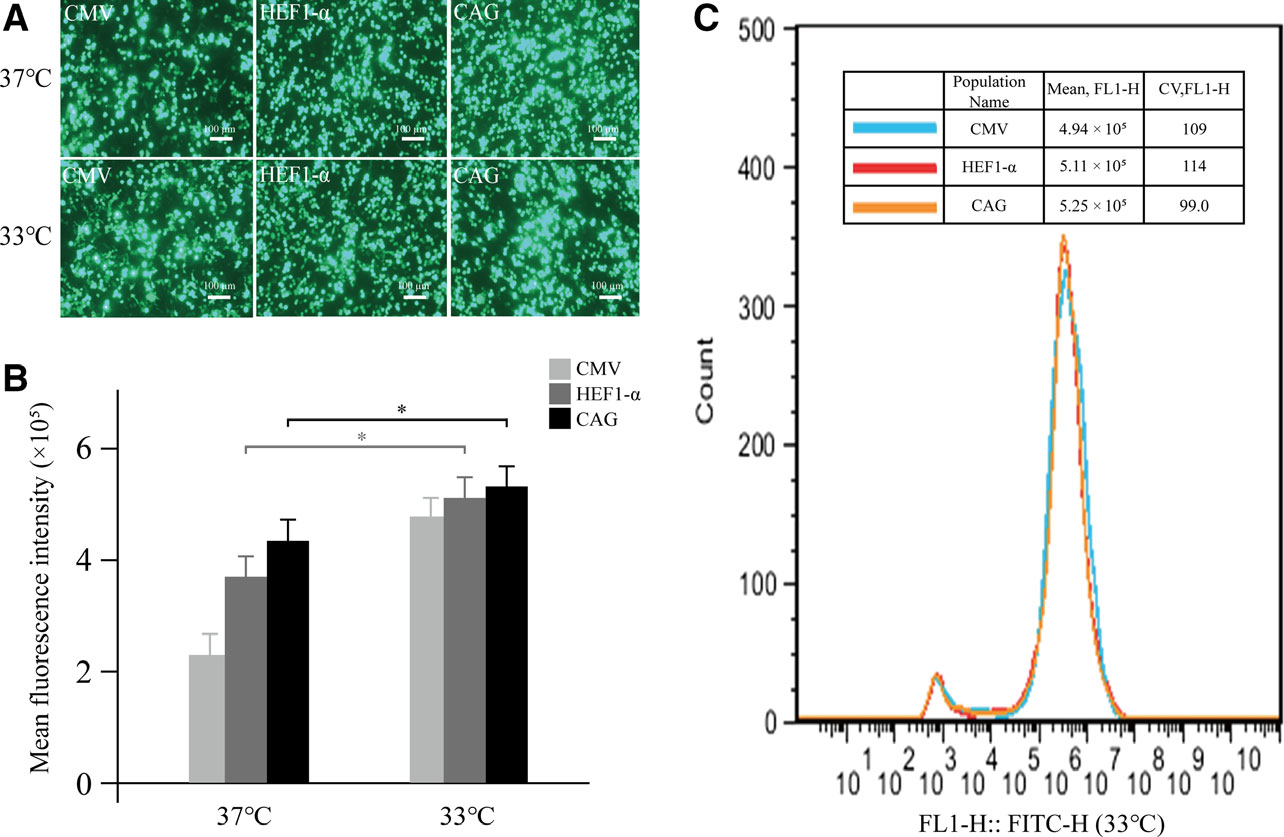
Effects of mild hypothermia on the expression of eGFP under different conditions. (A) Cells were transferred from 37 to 33 °C for 24 h and cultured for 7 days post‐transfection. The fluorescence intensity was observed under a fluorescence microscope, scale bars: 100 μm. (B) Cells were transferred from 37 to 33 °C for 7 days at 12, 24, and 48 h post‐transfection, and the expression levels of eGFP were detected by flow cytometry. (C) The eGFP MFI was determined by flow cytometry in a stably transfected cell pool with three highly expressed promoters, CAG, HEF‐α, and CMV mutant. The culture temperature was transferred from 37 to 33 °C 24 h after transfection. Cells were collected after 30 days, and eGFP fluorescence was measured by FACSCalibur. All the experiments were repeated three times. SEM is indicated (Student’s *t*‐test, *P < 0.05).

Next, we tested whether mild low‐temperature culture conditions could enhance eGFP expression. A previous study showed that reduced expression during the initial cell growth phase at low temperature (i.e., 33 °C) was followed by a boost in expression in production phase [29]. After transient transfection of the eGFP vectors at 37 °C, the cells were immediately transferred to 33 °C and incubated for 12, 24, and 48 h, while the control was maintained at 37 °C. Since the low‐temperature culture at 33 °C grew slower than the control culture at 37 °C, the low‐temperature culture was incubated for 24 h after transfection (Fig. 5). Fluorescence microscopy showed that changing the temperature from 37 to 33 °C led to an increase in the intensity of the green fluorescence in the cells. Flow cytometry showed that the protein expression levels were higher than those in control cells after 7 days. At 48 h after transfection, the culture temperature was changed from 37 to 33 °C, and eGFP expression decreased. Therefore, we believe that changing the incubation temperature from 37 to 33 °C at 24 h after transfection can promote the expression of eGFP.

### Expression of HBsAg

**Fig. 6:**
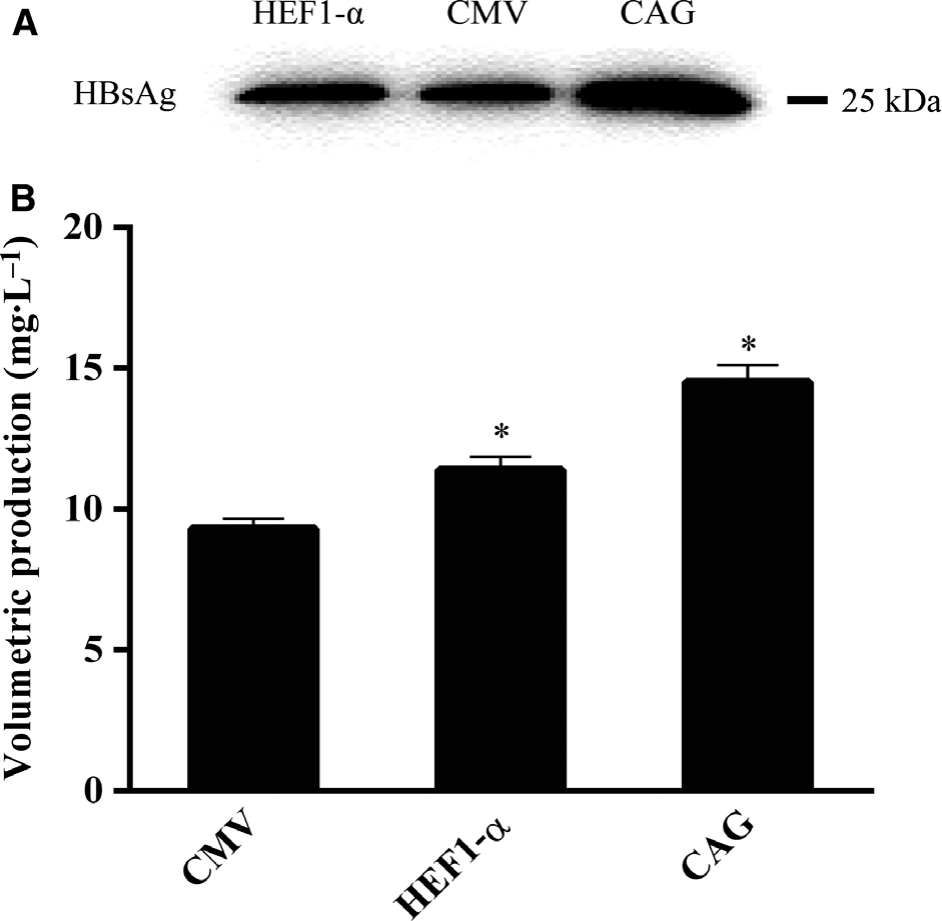
The expression of HBsAg protein. (A) The western blot of the HBsAg. (B) ELISA determined the yield of HBsAg. All the experiments were repeated three times. SEM is indicated (Student's *t*‐test, *P < 0.05).

To assess the influence of the promoter on the expression of a POI, we selected HBsAg and detected its expression in HEK293F cells in serum‐free medium. Western blotting confirmed that HBsAg expression driven by the CAG promoter was higher than that driven by the HEF‐1α promoter (Fig. 6A). ELISA showed that cells transfected with the CAG‐driven vector produced the highest HBsAg levels (14.5 mg·L^−1^; Fig. 6B), while cells transfected with the HEF‐1α‐driven vector produced 11.4 mg·L^−1^ HBsAg (Fig. 6B). Cells transfected with the control vector, containing the CMV promoter, produced 9.3 mg·L^−1^ HBsAg, indicating the protein yield can be increased significantly by changing the promoter component of the expression vector.

## Discussion

The promoter is a critical element in an expression vector, and selection of an optimal promoter can increase the expression and improve the stability of a gene of interest [39]. The CMV promoter is the most commonly used promoter for the production of recombinant proteins [40]. However, the CMV promoter has many potential methylation sites, which may lead to reduced recombinant protein production [41, 42]. In this study, the CMV promoter was used in the control vector to evaluate the transgene expression levels from seven other promoter‐driven vectors.

Of the eight promoters investigated here, the CAG enhancer was the shortest, followed by the mouse CMV and PGK promoters. Flow cytometry after transient transfection showed the highest eGFP gene expression levels from the CAG promoter but not the CAG enhancer. This indicated that the length of the promoter fragment is not the only factor affecting the efficiency of transfection. Since the CAG promoter vector had the highest transfection efficiency, in our opinion, the structure of the promoter itself is a key factor affecting transfection efficiency.

Cytomegalovirus is the most commonly used promoter for recombinant proteins, but due to presence of methylation site mutations, it lacks long‐term stability [43]. Therefore, it is crucial to find a more stable and efficient promoter. The HEF1‐α promoter can drive high levels of long‐term transgene expression in a lentiviral vector‐mediated system [44]. However, in the present study, it was not the most effective promoter. The results showed that among the eight tested commonly used promoters, the activity of the artificial CAG promoter construct was higher than the activity of the CMV and HEF‐α promoters in HEK93 cells. Expression levels of both the transient and stable eGFP transgenes driven by the CAG promoter were the highest, and the stability of the CAG vector was higher than that of the HEF1‐α and other promoter vectors.

Promoters are DNA elements that initiate the transcription of specific genes and are the key factors in determining the strength and time dynamics of transcription [28]. The results of the qRT‐PCR analysis showed that CAG drives the highest mRNA transcription levels in HEK93 cells, which were higher than the levels from other promoters such as HEF1‐α and CMV, and there was a linear relationship between eGFP mRNA and protein expression levels, indicating that the transgene expression levels are related to the transcription levels from the promoter vectors.

It has been reported that lowering the culture temperature to increase protein expression levels does not affect the function and activity of the recombinant protein [45]. We found that reducing the temperature of the culture from 37 to 33 °C at 24 h after transient transfection can effectively increase the expression level of recombinant proteins from promoter vectors in HEK293F cells. Flow cytometry showed that among the eight tested promoter vectors, low‐temperature treatment had the most obvious effect on transgene expression from the CMV promoter when compared with that of the control vector without low‐temperature treatment. However, the MFI of the CMV vector was still lower than that of the CAG vector. Based on this, we speculate that the CMV promoter was more temperature sensitive than the other promoters, but the CAG vector had the highest transgene expression level. Although low temperature can increase the expression levels of various recombinant proteins, it reduces the cell growth rate and inhibits cell division [46], so the culture temperature cannot be lower than 33 °C. In addition, the cells cannot be transferred to a low‐temperature environment too soon after transfection. This approach may be particularly useful when it is challenging to use chemical‐based expression methods for recombinant proteins.

To further examine the function of a strong promoter on a POI, we investigated the expression of HBsAg driven by the CAG and HEF1‐α promoters in serum‐free medium. The results indicated that the level of HBsAg produced by the CAG promoter was markedly higher than that produced by the CHEF1‐α promoter in HEK293 cells, further confirming the effects of the CAG promoter.

In conclusion, we first identified the impact of the CAG promoter on the expression of recombinant proteins as both transient and stable transgenes in HEK293F cells. Then, we found that a mild low temperature (i.e., 33 °C) increased the activity of the CMV and CAG promoters in HEK293F cells and increased recombinant protein productivity.

## Conflict of interest

The authors declare no conflict of interest.

## Author contributions

T‐YW and XW conceived and designed the project, Y‐YD acquired the data, Y‐LJ and C‐PZ analyzed and interpreted the data, Y‐YD and YL wrote the paper.

## Data Availability

Data and details of the analyses are available from the corresponding author upon reasonable request.
